# Effectiveness of Interventions to Improve Cardiovascular Perturbations in Women with Exercise-Associated Amenorrhea: A Systematic Review

**DOI:** 10.1177/26884844251379434

**Published:** 2025-09-18

**Authors:** Nicole L. Tegg, Jenna Semmens, Emma O’Donnell, Caitlynd Myburgh, Ashley Hyde, Megan Kennedy, Colleen M. Norris

**Affiliations:** ^1^ Faculty of Nursing, University of Alberta, Edmonton, Alberta, Canada.; ^2^ School of Sport, Exercise and Health Sciences, Loughborough University, Leicestershire, UK.; ^3^ Faculty of Natural Sciences, The Kings University, Edmonton, Alberta, Canada.; ^4^ University of Alberta Library, Edmonton, Alberta, Canada.; ^5^ Cavarzan Chair in Women’s Research, WCHRI, Edmonton, Alberta, Canada.; ^6^ Faculty of Medicine, School of Public Health Sciences, University of Alberta, Edmonton, Alberta, Canada.

**Keywords:** cardiovascular health, endothelial function, exercise, athletic amenorrhea, women

## Abstract

**Objectives:**

Women with exercise-associated amenorrhea demonstrate cardiovascular perturbations such as endothelial dysfunction and altered lipid profiles. The objective of this systematic review was to assess the effectiveness of pharmacological/nutraceutical and non-pharmacological interventions for improving these cardiovascular perturbations.

**Design, Data Sources, and Eligibility Criteria:**

A literature search was performed in October 2023 and updated in July 2024 of CINAHL (EBSCOhost), Cochrane Library, Embase (Ovid), MEDLINE (Ovid), SPORTDiscus (EBSCOhost), and Scopus from inception to present with no date or language limitations and four sources of gray literature. Experimental and quasi-experimental pre–post studies of women with exercise-associated amenorrhea, using pharmacological/nutraceutical or non-pharmacological intervention, were included.

**Results and Summary:**

Three studies from three countries were included. Interventions included 9 months of low-dose oral contraceptives and 4 weeks of folic acid (10 mg/day). Both interventions improved endothelial function in women experiencing exercise-associated amenorrhea, from 1.42% to 4.88% and 3.0% to 7.7%, respectively. The impact of oral contraceptives on lipids was conflicting, and increases were seen in select inflammatory markers, including high-sensitivity C-reactive protein and tumor necrosis factor.

**Conclusion:**

Oral contraceptives or folic acid may improve the endothelial dysfunction associated with exercise-associated amenorrhea. As cardiovascular disease remains a global cause of mortality for women, further investigation into the long-term cardiovascular consequences of impaired vascular and lipid profiles of exercise-associated amenorrhea is warranted.

## Introduction

Women remain under-researched for cardiovascular disease (CVD), and efforts to improve cardiovascular health in women have stagnated over the last decade.^1^ It is important to consider less recognized possible risk factors for CVD in women, such as secondary amenorrhea. Secondary amenorrhea is characterized by cessation of menses for at least three consecutive months and estrogen deficiency.^2^ A common form of secondary amenorrhea is functional hypothalamic amenorrhea (FHA), which is estimated to affect 17.4 million women globally,^3^ accounting for about one-third of all cases of secondary amenorrhea.^2,4^ There are three interrelated, often overlapping pathogeneses of FHA, including exercise-associated (EAA), weight-loss or stress-related.^5^ While the prevalence is 3%−14% in the general population,^6–8^ it rises to 40%−56% in recreational and competitive endurance athletes (*i.e.,* running, triathlon, cycling)^9–13^ and in women working in physically demanding occupational jobs.^14–16^ Studies of women with exercise-associated FHA report similar weights, body compositions, and BMIs compared with their eumenorrheic counterparts. This suggests that despite chronic energy deficiency and high energy expenditure, athletes with exercise-associated FHA remain of similar, not lower, body weight and composition as energy-replete, regularly menstruating women.^17,18^ Given the high prevalence of FHA in exercising and physically active women, this review focuses on FHA due to EAA.

Women in their reproductive years are considered to be at a lower risk of CVD when compared with age-matched men.^19^ For men and women, impaired endothelial function is a permissive factor in the development of atherosclerosis.^20,21^ Regular aerobic exercise training attenuates the age-associated decline in endothelial function.^22^ However, sex differences in this finding have been reported. Aerobic exercise augmented endothelial function in middle-aged men compared with untrained men.^23^ In contrast, estrogen-deficient, trained postmenopausal women demonstrated endothelial function similar to untrained postmenopausal women, but estradiol therapy was shown to augment endothelial function in response to endurance training.^24^ This suggests that estrogen may be essential in conferring endothelial adaptations to exercise in women.^24^

In the vasculature, endogenous estrogen is essential in maintaining vessel integrity and vascular function. Estrogen increases the production and bioavailability of endothelial nitric oxide *via* the phosphoinositide 3-kinase/protein kinase B pathway.^25^ Nitric oxide, a potent vasodilator, plays an essential role in vascular health, facilitating a quiescent state of the vascular wall by inhibiting inflammation, platelet aggregation, and vascular smooth muscle cell adhesion and proliferation.^26,27^ Accordingly, estrogen deficiency is associated with decreased endothelium-derived nitric oxide production^28,29^ and impaired vascular function.^30,31^ Estrogen has also been shown to have numerous anti-inflammatory effects.^32,33^

A recent systematic review and meta-analyses^34^ found that compared with age- and fitness-matched controls with eumenorrhea, women experiencing EAA demonstrated significantly lower estrogen levels. The EAA women demonstrated significantly reduced flow-mediated dilation (FMD), suggesting the beneficial effects of exercise may be obviated in these women.^35,36^ The EAA women also demonstrated adverse lipid profile changes, including significantly higher total cholesterol, low-density lipoprotein cholesterol, and triglycerides. These findings are consistent with lipid profiles in postmenopausal women.^37^ Lastly, nonsignificant differences were found between groups for oxidative stress and apolipoproteins. It is not known if the cardiovascular perturbations of EAA present a potential future cardiovascular health burden. Therefore, we sought to synthesize the evidence seeking to improve the cardiovascular perturbations in women experiencing EAA.

A preliminary search of the Cumulative Index to Nursing and Allied Health Literature, the Cochrane Database of Systematic Reviews, *Joanna Briggs Institute (JBI) Evidence Synthesis*, MEDLINE and the International Prospective Register of Systematic Reviews (PROSPERO) did not reveal any completed or ongoing systematic review on the topic of improving the cardiovascular perturbations of EAA. This systematic review aims to determine if the cardiovascular perturbations of EAA (*e.g.*, endothelial dysfunction, lipid profiles, inflammatory markers, *etc.*) can be improved. Our results may be important for the prevention of CVDs in women.

### Review question

What is the effectiveness of non-pharmacological and pharmacological interventions to improve the cardiovascular perturbations of EAA?

## Methods

As we used previously published anonymized data, informed consent or approval from an institutional review board was not required. The JBI methodology for systematic reviews of effectiveness was followed in the conduct of this review and synthesis.^38^ The review was conducted in accordance with an *a priori* protocol registered with PROSPERO (CRD42023483856). The *JBI* standardized data extraction and critical appraisal tools are publicly available in *JBI SUMARI* and in the *JBI Manual for Evidence Synthesis.*

### Inclusion criteria

#### Participants

This review included studies that included exercise-trained women experiencing EAA. The authors acknowledge that amenorrhea is experienced by those whose biological sex assigned at birth is “female,” but they may not identify as cisgender; recognizing language inconsistencies in the literature, we included studies that described their sample as females or women. We considered “exercising” as purposeful exercise for a minimum of 2 hours per week and less than 2 hours per week as sedentary^39^ and EAA as the absence of menses for a minimum of three consecutive cycles.^2^ Due to variability in the ages of experiencing menarche, no lower age limit was applied.^40^ To reflect the age of early natural menopause, we set an upper age limit of 45 years.^41^

#### Intervention

This review considered studies that used pharmacological/nutraceutical interventions (*e.g.*, oral contraceptive pills [OCP], folic acid) or non-pharmacological interventions (*e.g.*, increased caloric intake, decreased training time/frequency, resumption of menses).

#### Comparator

This review considered any pre–post comparison of a pharmacological/nutraceutical or non-pharmacological intervention for EAA against a control group.

#### Outcomes

The primary outcome of interest was endothelial function, as it is a sentinel event in the development of atherosclerosis and can predict future cardiovascular events.^20,21^ The secondary outcomes included additional cardiovascular perturbations of EAA (lipid profiles, inflammatory markers, arterial stiffness, or changes to cardiovascular physiology). Studies were excluded if no cardiovascular data were reported.

### Types of studies

This review considered experimental and quasi-experimental study designs, including randomized controlled trials, non-randomized controlled trials, pre–post studies, and interrupted time-series studies. We excluded non-research-based designs.

### Search strategy

This search is reported according to the PRISMA-S extension for searching,^42^ and methodological guidance for searching was sought from the Cochrane Handbook of Systematic Reviews of Interventions, Chapter 4: Searching for and Selecting Studies.^43^ The search strategy aimed to locate published and unpublished studies. A preliminary search was performed in MEDLINE (Ovid) on October 17, 2023. The text words in the titles and abstracts of relevant articles and the index terms were used in developing the full search strategy for MEDLINE (Ovid). The comprehensive and systematic search strategy was developed collaboratively with an experienced health sciences librarian (M.K.) to identify all relevant published studies.

The following bibliographic databases were searched from inception to November 15th, 2023: Medline (1946 to Present) and EMBASE (1974 to Present) *via* OVID; Cumulative Index to Nursing and Allied Health Literature-CINAHL (1936 to Present); SPORTDiscuss (inception to present) *via* EBSCOhost; Scopus (1976 to Present) *via* Elsevier; and Cochrane Library (1993 to Present) *via* Wiley. Databases were searched using natural language keywords and subject headings, such as Medical Subject Headings, wherever they were available. The search strategy was constructed using the Population, Intervention, Comparison, Outcome model: (1) women with FHA or menstrual disturbances who are athletes, physically fit, or exercise frequently; (2) CVDs; (3) pharmacological/nutraceutical and non-pharmacological interventions. To increase search sensitivity, no publication date or language limits were applied to the search results. Non-peer-reviewed materials such as conference proceedings, letters, opinions, and books were removed from the results. Search results were updated in July 2024. Four sources of gray literature were searched. The complete search strategy is found in Supplementary Data S2.

### Study selection

**FIG. 1.**
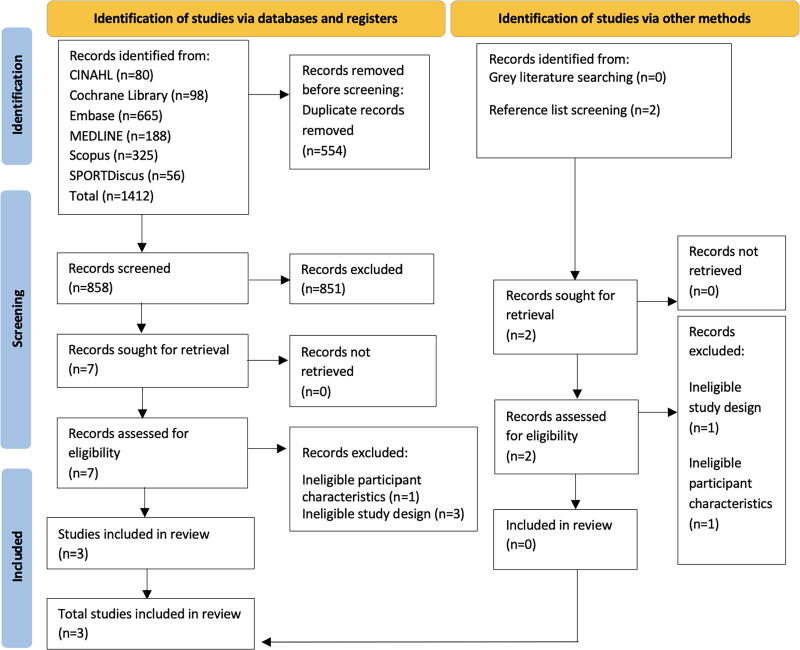
PRISMA flow diagram. Search results and study selection and inclusion process.^45^ PRISMA, preferred reporting items for systematic reviews and meta-analyses.

Following the systematic search, the identified citations were collated and uploaded to Endnote V20.4 (Clarivate Analytics, PA, USA), and duplicates were removed manually. Citations were uploaded into the JBI System for the Unified Management, Assessment, and Review of Information (JBI SUMARI; Joanna Briggs Institute, Adelaide, Australia).^44^ Two independent reviewers (N.L.T., J.S.) performed all screening stages. A pilot test was performed with 7% (50) of the titles and abstracts. As one reviewer’s responses were a constant, we were unable to calculate Cohen’s kappa; reviewer agreement was 96%. Titles and abstracts were then screened against *a priori* inclusion and exclusion criteria. The full texts of potentially eligible studies were retrieved and assessed in detail against the inclusion/exclusion criteria. At each stage of screening, disagreements were resolved through discussion. The search and study selection and inclusion process results are reported using a Preferred Reporting Items for Systematic Reviews and Meta-analyses (PRISMA) flow diagram (Fig. 1).^45^

### Assessment of methodological quality

Two independent reviewers (N.L.T., J.S.) critically appraised the selected studies for methodological quality using standardized JBI critical appraisal instruments.^38^ Both reviewers discussed the results in a final appraisal in accordance with the JBI methodology.^38^ Disagreements were resolved through discussion. We determined the level of methodological quality as fair if less than 50% of the items were rated “yes,” moderate if between 51% and 80% of the items were rated “yes,” and good if more than 80% of the items were rated “yes.” All studies were included regardless of methodological quality.

### Data extraction and synthesis

Two independent reviewers (N.L.T., J.S.) extracted data using the standardized data extraction tool from JBI SUMARI,^46^ following a pilot test with one study to ensure both reviewers were extracting sufficient data. The extracted data included details about the study setting, participant characteristics and groups, intervention, comparator, outcomes, methods, and results. Disagreements were resolved by discussion. Due to the differences in study groups and interventions, a meta-analysis was not performed, and the data are described narratively.

## Results

### Study inclusion

A total of 1414 records were retrieved through database searching and citation chaining. After removing duplicates, 858 records were screened by title and abstract for eligibility. Of the remaining records, nine full-text articles were retrieved and assessed for eligibility. Six articles were excluded; articles with reasons for exclusion are provided in Supplementary Table S1. The remaining three studies were included and critically appraised.

### Methodological quality

Three studies were assessed for methodological quality. The results of the critical appraisals are found in Supplementary Tables S2 and S3. One study was of fair quality^47^ and two were of good quality.^48,49^ All studies provided reasons for exclusions or withdrawals. Potential confounding causes of secondary amenorrhea, such as thyroid disease and pituitary tumors, were addressed by way of inclusion/exclusion criteria in the individual studies. All the studies included exercise-trained women. One study compared EAA with exercising women with eumenorrhea and a sedentary control group.^48^ One compared EAA with exercising women with eumenorrhea,^49^ and one compared two groups of EAA (OCP vs. a control group of Calcium and Vitamin D).^47^

### Characteristics of included studies

The three included study designs were a single-blind randomized clinical trial^47^ and two pre–post quasi-experimental studies.^48,49^ Characteristics of the included studies are summarized in Table 1. The studies included in this review were published between 2005 and 2018 in three countries, Iran,^47^ Sweden,^48^ and the United States,^49^ and were conducted in medical centers. The total number of participants in all studies was 69, and the number of participants per study ranged from 13 to 36. The population consisted of exercise-trained women participating in various types of endurance training. The inclusion/exclusion criteria were clearly defined, requiring participants to be physically active, healthy, free of preexisting disease, and not taking hormonal therapy such as OCPs. The identified interventions included folic acid 10 mg per day for 4 weeks^49^ and 9 months of low-dose monophasic, combined OCP (30 µg ethinyl estradiol and 150 µg levonorgestrel).^47,48^ No non-pharmacological/nutraceutical interventions, such as lifestyle modifications, were identified that were eligible for this review. However, there is some evidence showing that endothelial function can be improved with the resumption of menses.^50,51^ Outcomes were measured reliably, and two studies^48,49^ used appropriate statistical analysis. Two studies considered EAA to be the absence of menses for three or more consecutive cycles,^48,49^ and one required participants to experience EAA for at least 2 years.^47^ Primary outcomes included endothelial function,^48,49^ bone mineral density, and cardiovascular perturbations (lipid profiles, inflammatory markers, *etc.*).^47^

**Table 1.** Characteristics of Included Studies

Study	Country and setting	Participant characteristics	Groups	Intervention	Outcomes measured	Main findings
Dadgostar et al., 2018^47^ Single-blind randomized clinical trial	Iran Clinical Research Hospital	18 Iranian elite female athletes with a min 2-yr history of FHA 13 included in analysis	10 OCP: BMI 21.72 (1.60) 8 control group: BMI 22.06 (3.59)	OCP group: low dose combined OCP (30 µg ethinyl estradiol + 150 µg levonorgestrel), 1000 mg/d calcium, 400 IU/d vitamin D for 9 mo Control group: 1000 mg/d calcium and 400 IU/d vitamin D for 9 mo	Spine and femur bone density, lipids, inflammatory markers, blood glucose	No changes to bone density, some positive changes noted in lipid profiles, increase in one inflammatory marker.
Hoch et al., 2010^49^ Pre–post quasi-intervention	USA Academic Medical Center	20 White women FHA: absence of 3 or more consecutive menses Running min 20 miles/wk for min 1 yr	7 AA: age 22.1 (4.8), BMI 21.0 (2.8), age at menarche 13.9 (1.6), running miles/wk 32.5 (13.5) 10 EA: age 27.2 (6.3), BMI 21.9 (2.9), age at menarche 12.3 (1.5), running miles/wk 32 (8.9)	Folic acid 10 mg/d for 4 wks	FMD, HR, BP	Folic acid improved FMD in AAs.
Rickenlund et al., 2005^48^ Pre–post quasi-intervention	Sweden University Hospital	36 women FHA: absence of 3 or more consecutive menses Training min 6 h aerobic weight-bearing training of the legs/wk or minimum 70 km running/wk for at least 6 mo	11 AA: age 19.5 (5.1), BMI 18.6 (1.1), age at menarche 14.0 (1.4), training hrs/wk 7.5 (1.0) Thirteen EA: age 21.4 (4.0), BMI 19.7 (1.3), age at menarche 12.2 (1.2), training hrs/wk 7.7 (1.8) 12 Sedentary controls: Age 20.9 (4.2), BMI 19.6 (1.6), age at menarche 13.1 (1.4)	Low dose, monophasic, combined OC (30 µg ethinyl estradiol + 150 µg levonorgestrel d 1–21, hormone-tablet-free interval on d 22–28) for 9 mo	FMD, HR, BP, lipids, inflammatory markers	OCP improved FMD, moderate unfavourable change in lipid profiles was seen in all three groups. Increase in select inflammatory markers.

Values are mean (SD). AA, athletes with amenorrhea; BP, blood pressure; EA, athletes with eumenorrhea; FHA, functional hypothalamic amenorrhea; FMD, flow-mediated dilation; HR, heart rate; OCP, oral contraceptive pills.

### Review findings

#### Endothelial function

Rickenlund et al.^48^ reported that following 9 months of OCP use, FMD% significantly increased from 1.42% to 4.88% in EAA and sedentary controls from 4.59% to 7.01%, while a nonsignificant difference was noted in exercising women with eumenorrhea from 6.59% to 5.25%. Hoch et al.^49^ found a significant increase in FMD% from 3.0% to 7.7% in EAA when treated with folic acid for 4 weeks, while FMD% remained statistically similar in exercising women with eumenorrhea (6.7%–5.9%).

#### Lipid profiles

Rickenlund et al.^48^ reported that lipid profiles did not differ between amenorrheic and eumenorrheic athletes before or after 9 months of OCP use. Within groups, high-density lipoprotein significantly decreased in all groups. Dadgostar et al.^47^ found that after 9 months of OCP, very low-density lipoprotein, Apo B, and Apo B/Apo A ratio were decreased in athletes who were amenorrheic at study entry. No differences were found in high-density lipoprotein, triglycerides, or Apo A. See Table 2.

**Table 2.** Lipid Profiles

Measure	AAs pre	AAs post	EAs pre	EAs post	Controls pre	Controls post
Triglycerides (mg/dL)						
Rickenlund-OCs	79 ± 30	96 ± 27	77 ± 32	93 ± 33	78 ± 39	77 ± 17
Dadgostar-OCs	69.29 ± 18.52	69.62 ± 21.99	N/A	N/A	98.80 ± 55.26	78.4 ± 39.49
Cholesterol (mg/dL)						
Rickenlund-OCs	185 ± 19	181 ± 39	165 ± 23	169 ± 27	177 ± 27	173 ± 27
Dadgostar-OCs	191.86 ± 41.55	170.75 ± 38.01	N/A	N/A	159.60 ± 17.57	151.80 ± 26.32
LDL (mg/dL)						
Rickenlund-OCs	115 ± 19	115 ± 27	100 ± 19	108 ± 23	108 ± 23	112 ± 23
Dadgostar-OCs	86.43 ± 18.72	101.12 ± 27.94	N/A	N/A	78.20 ± 17.57	83.80 ± 31.40
HDL (mg/dL)						
Rickenlund-OCs	51 ± 8	47 ± 6	51 ± 4	47 ± 5	55 ± 9	43 ± 5
Dadgostar-OCs	50.86 ± 10.37	46.87 ± 6.96	N/A	N/A	51.0 ± 11.98	50.40 ± 11.89
VLDL (mg/dL)						
Dadgostar-OCs	51.51 ± 22.49	22.75 ± 13.59	N/A	N/A	29.75 ± 18.82	17.60 ± 7.96
Apo B/Apo A						
Dadgostar-OCs	0.63 ± 0.19	0.44 ± 0.14	N/A	N/A	0.62 ± 0.21	0.37 ± 0.13
Lp(a) g/L						
Rickenlund -OCs	0.09 (0.02–0.12)	0.06 (0.02–0.10)	0.19 (0.08–0.26)	0.21 (0.07–0.28)	0.08 (0.02–0.23)	0.06 (0.02–0.21)
Apo A						
Rickenlund-OCs	154 ± 15	152 ± 17	155 ± 12	153 ± 10	157 ± 21	142 ± 14
Dadgostar-OCs	162.17 ± 46.31	166.25 ± 23.74	N/A	N/A	113.18 ± 20.31	162.60 ± 14.81
Apo B						
Rickenlund-OCs	89 ± 12	103 ± 28	77 ± 15	95 ± 21	81 ± 19	99 ± 20
Dadgostar-OCs	91.40 ± 11.87	67.25 ± 16.46	N/A	N/A	68.06 ± 20.23	59.40 ± 18.58

Values are mean ± SD or median (P_25_−P_75_). Legend: Apo A, apolipoprotein A; Apo B, apolipoprotein B; HDL, high-density lipoprotein cholesterol; LDL, low-density lipoprotein cholesterol; VLDL, very low-density lipoprotein cholesterol.

#### Inflammatory markers

Rickenlund et al.^48^ reported an overall significant increase in high-sensitivity C-reactive protein and tumor necrosis factor-α within, but not between groups following 9 months of OCP use. These authors also reported an overall significant decrease within, but not between, groups in vascular cell-adhesion molecule-1. Dadgostar et al.^47^ reported no significant differences in inflammatory markers after 9 months of OCP use. See Table 3.

**Table 3.** Inflammatory Markers

Measure	AAs pre	AAs post	EAs pre	EAs post	Controls pre	Controls post
hsCRP (mg/L)						
Rickenlund-OCs	0.19 (0.09–0.88)	0.75 (0.41–1.16)	0.45 (0.34–0.88)	1.39 (0.81–1.57)	0.40 (0.14–1.07)	1.14 (0.80–3.60)
IL-6 (pg/mL)						
Rickenlund-OCs	0.55 (0.48–0.77)	0.84 (0.46–1.23)	0.97 (0.69–1.98)	0.71 (0.53–1.14)	1.25 (0.76–1.68)	1.16 (0.71–1.48)
TNF-α (pg/mL)						
Rickenlund-OCs	1.4 (1.1–7.5)	1.4 (1.2–11.1)	1.4 (1.1–2.3)	1.6 (1.4–2.1)	1.5 (0.9–2.7)	1.5 (0.8–3.1)
VCAM-1 (ng/mL)						
Rickenlund-OCs	500 ± 95	428 ± 91	483 ± 66	390 ± 53	526 ± 92	422 ± 112
Homocysteine						
Dadgostar-OCs	10.13 ± 2.44	8.50 ± 1.99	N/A	N/A	11.50 ± 2.96	8.30 ± 0.57
Fibrinogen						
Dadgostar-OCs	209.20 ± 32.76	252.0 ± 46.27	N/A	N/A	219.80 ± 52.22	248.0 ± 37.20

Values are mean ± SD or median (P_25_–P_75_). Legend: hsCRP, high-sensitivity C-reactive protein; IL-6, interleukin 6; TNF-α, tumor necrosis factor-alpha; VCAM-1, Vascular cell adhesion protein-1.

## Discussion

In this review, we sought to identify the effectiveness of pharmacological/nutraceutical interventions that may improve the cardiovascular perturbations in vascular and lipid profiles observed in women with EAA. The identified outcomes included endothelial dysfunction, lipid profiles, and inflammatory markers. The evidence in this area is limited. This systematic review identified one experimental and two quasi-experimental studies. The identified interventions included folic acid 10 mg per day for 4 weeks^49^ and 9 months of low-dose monophasic, combined OCP (30 µg ethinyl estradiol and 150 µg levonorgestrel).^47,48^

There were some conflicting findings on the effect of OCPs on lipid profiles in EAA. This may have been partly due to small sample sizes, and it is unclear if correction for multiple comparisons was performed by Dadgostar et al.^47^ Increases were observed in the inflammatory markers, high-sensitivity C-reactive protein, and tumor necrosis factor-α. Importantly, both folic acid supplementation and OCPs lead to improvements in endothelial function. The exact mechanisms of action are unclear. The improvements in endothelial function from folic acid are postulated to be due to the regeneration of tetrahydrobiopterin, a cofactor in the nitric oxide synthase production of endothelium-derived nitric oxide.^52,53^ In OCPs, the mechanism is thought to be the estrogenic component, as estrogen increases the bioavailability of nitric oxide.^54,55^

The findings on improvements to endothelial dysfunction are consistent with evidence exploring the use of estrogen replacement in estrogen-deficient pre- and postmenopausal women. Exogenous estrogen supplementation has been shown to improve endothelial function in premenopausal women who have undergone bilateral oophorectomy and hysterectomy^56^ and to positively affect the endothelium in postmenopausal women.^57,58^ Folic acid supplementation has also been shown to exert variable effects on endothelial function in eumenorrheic exercising women. For example, in eumenorrheic runners, 6 weeks of 10 mg per day of folic acid significantly increased FMD values by 3.5%.^59^ In contrast, 4 weeks of folic acid at 10 mg per day did not statistically alter FMD in eumenorrheic runners,^49^ suggesting a longer duration of supplementation of folic acid may be needed to help bring about improvements in FMD. However, in eumenorrheic, oligomenorrheic, and amenorrheic professional ballet dancers who presented with reduced FMD (<5%) at study entry, 4 weeks of 10 mg per day of folic acid significantly increased FMD from ∼3% to 7%,^60^ suggesting that folic acid may exert favorable vascular effects in women who present with impaired FMD, regardless of menstrual status.

While treatment with folic acid and OCP may improve endothelial function, neither addresses the underlying cause of EAA nor the consequences of energy deficiency in women with FHA. Non-pharmacological treatment of FHA should be the prioritized treatment strategy, including increased energy availability, weight gain, and resumption of normal menses.^61^ Regular menstruation and estrogen levels are important to bone health^62–65^ and confer cardioprotective effects.^26,54,61,66^ It is important to note that there may be challenges to restoring energy balance (and subsequently menstruation) in women with FHA due to complex factors, including concerns about leanness or body image, athletic performance, and patterns of disordered eating.^10^ The withdrawal bleeding associated with OCPs is not a restoration of menses but rather a result of exogenous hormones and can create a false sense of security.

Women may choose to use estrogen-containing OCPs for numerous reasons, such as contraception, management of painful periods, menstrual migraines, premenstrual syndrome, or acne.^67^ In exercising women with FHA, estrogen-containing OCPs may be administered to protect bone health in those with very low bone mineral density.^61^ However, OCP use is not consistently associated with improvements in bone mineral density in endurance athletes.^68,69^ Negative effects of OCPs on bone health are associated with the “first pass effect,” whereby ethinyl estradiol metabolism in the liver decreases hepatic synthesis of Insulin-like growth factor I (IGF-I).^70^ IGF-I is an important stimulus for bone formation. With consideration of contraceptive needs and contraindications, the current recommendation for women with FHA requiring pharmacological therapy is hormone replacement therapy consisting of transdermal estrogen with cyclic progesterone.^61^ Common side effects of combined OCP use include regular vaginal bleeding, nausea, bloating, sore or tender breasts, and headaches.^71^ Hypertension may also occur in ∼4% to 5% of women.^72^ OCPs have also been associated with a 1.6-fold increased risk of myocardial infarction or ischemic stroke (risk is highest in OCPs containing >50µg of estrogen)^73^ and a small absolute increased risk of venous thrombosis, particularly during the first year.^74^ Low-dose OCPs contain less than 50 µg of estrogen, with high-dose OCPs containing 50 µg or greater.^75^ Currently, low-dose OCPs are the preferred formulation.^72^

As CVD remains the leading cause of death for women globally,^1^ it is important to consider strategies to increase the reporting and, thereby, the subsequent recognition and treatment of FHA in women. Beyond the cardiovascular perturbations, FHA is associated with deleterious effects on bone mineral density and changes to metabolic hormone profiles and reproductive hormones.^2^ While it is positive to note that endothelial function can be improved in EAA with the resumption of menses,^50,51^ the long-term cardiovascular consequences of FHA in this population of women remain unknown. Importantly, the clinical significance of these findings requires larger, long-term studies.

## Study Limitations

This review has several limitations. One included study^47^ had several methodological flaws that may affect the validity of the findings. Furthermore, the limited evidence makes it challenging to determine the magnitude of effectiveness of the identified interventions, as a meta-analysis could not be performed. Due to our inclusion and exclusion criteria, we cannot generalize our findings to women beyond those with EAA. It is important to note there was heterogeneity between studies for the diagnostic criteria of FHA and the type and duration of exercise training. Lastly, as the included studies had small sample sizes, the risk for type II errors is high.

## Conclusions

Cardiovascular disease presents a global health burden for women, remaining a leading cause of mortality. It is imperative to begin considering the impact of less recognized risk factors for CVD that may occur during the reproductive years for women, such as FHA. Due to the limited number of studies examining interventions to improve these cardiovascular perturbations of EAA, a meta-analysis for the magnitude of the effectiveness of these interventions cannot be performed at present. However, it appears that the endothelial dysfunction may be improved with hormonal contraceptives and folic acid supplementation. As the long-term cardiovascular effects of hypoestrogenemia in athletic women of reproductive age are unknown, future prospective studies are warranted. Lastly, increasing awareness in women and clinicians of the adverse cardiovascular alterations associated with energy deficiency is urgently needed.

## Data Availability

The authors declare that all supporting data are available within this article and supplemental file. The Preferred Reporting Items for Systematic Reviews and Meta-Analyses checklist is available in Data S1.

## Patient and Public Involvement

Patients and/or the public were not involved in the design, or conduct, or reporting, or dissemination plans of this research.

## Abbreviations Used

BMI: body mass index
CVD: cardiovascular disease
EAA: exercise-associated amenorrhea
FHA: functional hypothalamic amenorrhea
FMD: flow-mediated dilation
JBI: Joanna Briggs Institute
OCP: oral contraceptive pills
PRISMA: Preferred Reporting Items for Systematic Reviews and Meta-analyses
