# Supplementary material for: Effectiveness of Interventions to Improve Cardiovascular Perturbations in Women with Exercise-Associated Amenorrhea: A Systematic Review
Source: Womens Health Rep (New Rochelle). 2025 Sep 18;6(1):925–35. doi: 10.1177/26884844251379434 (PMC12547389; doi:10.1177/26884844251379434)
Supplement: Supplementary Data [file 26884844251379434_supplementary_data.docx]

**Data S1:** PRISMA 2020 Checklist

| **Section and Topic** | **Item #** | **Checklist item** | **Location where item is reported** |
| --- | --- | --- | --- |
| **TITLE** | | |  |
| Title | 1 | Identify the report as a systematic review. | P1 |
| **ABSTRACT** | | |  |
| Abstract | 2 | See the PRISMA 2020 for Abstracts checklist. | P 2 |
| **INTRODUCTION** | | |  |
| Rationale | 3 | Describe the rationale for the review in the context of existing knowledge. | P 3-4 |
| Objectives | 4 | Provide an explicit statement of the objective(s) or question(s) the review addresses. | P 4 |
| **METHODS** | | |  |
| Eligibility criteria | 5 | Specify the inclusion and exclusion criteria for the review and how studies were grouped for the syntheses. | Pg 4-5 |
| Information sources | 6 | Specify all databases, registers, websites, organisations, reference lists and other sources searched or consulted to identify studies. Specify the date when each source was last searched or consulted. | Pg 5 |
| Search strategy | 7 | Present the full search strategies for all databases, registers and websites, including any filters and limits used. | Suppl. |
| Selection process | 8 | Specify the methods used to decide whether a study met the inclusion criteria of the review, including how many reviewers screened each record and each report retrieved, whether they worked independently, and if applicable, details of automation tools used in the process. | P 6 |
| Data collection process | 9 | Specify the methods used to collect data from reports, including how many reviewers collected data from each report, whether they worked independently, any processes for obtaining or confirming data from study investigators, and if applicable, details of automation tools used in the process. | P 6 |
| Data items | 10a | List and define all outcomes for which data were sought. Specify whether all results that were compatible with each outcome domain in each study were sought (e.g. for all measures, time points, analyses), and if not, the methods used to decide which results to collect. | P 5 |
|  | 10b | List and define all other variables for which data were sought (e.g. participant and intervention characteristics, funding sources). Describe any assumptions made about any missing or unclear information. | P 6 |
| Study risk of bias assessment | 11 | Specify the methods used to assess risk of bias in the included studies, including details of the tool(s) used, how many reviewers assessed each study and whether they worked independently, and if applicable, details of automation tools used in the process. | P 6 |
| Effect measures | 12 | Specify for each outcome the effect measure(s) (e.g. risk ratio, mean difference) used in the synthesis or presentation of results. | N/A |
| Synthesis methods | 13a | Describe the processes used to decide which studies were eligible for each synthesis (e.g. tabulating the study intervention characteristics and comparing against the planned groups for each synthesis (item #5)). | N/A |
|  | 13b | Describe any methods required to prepare the data for presentation or synthesis, such as handling of missing summary statistics, or data conversions. | N/A |
|  | 13c | Describe any methods used to tabulate or visually display results of individual studies and syntheses. | N/A |
|  | 13d | Describe any methods used to synthesize results and provide a rationale for the choice(s). If meta-analysis was performed, describe the model(s), method(s) to identify the presence and extent of statistical heterogeneity, and software package(s) used. | N/A |
|  | 13e | Describe any methods used to explore possible causes of heterogeneity among study results (e.g. subgroup analysis, meta-regression). | N/A |
|  | 13f | Describe any sensitivity analyses conducted to assess robustness of the synthesized results. | N/A |
| Reporting bias assessment | 14 | Describe any methods used to assess risk of bias due to missing results in a synthesis (arising from reporting biases). | N/A |
| Certainty assessment | 15 | Describe any methods used to assess certainty (or confidence) in the body of evidence for an outcome. | N/A |
| **RESULTS** | | |  |
| Study selection | 16a | Describe the results of the search and selection process, from the number of records identified in the search to the number of studies included in the review, ideally using a flow diagram. | P 6 Fig 1 |
|  | 16b | Cite studies that might appear to meet the inclusion criteria, but which were excluded, and explain why they were excluded. | Table S1 |
| Study characteristics | 17 | Cite each included study and present its characteristics. | Table 1 |
| Risk of bias in studies | 18 | Present assessments of risk of bias for each included study. | Tables S2 & S3 |
| Results of individual studies | 19 | For all outcomes, present, for each study: (a) summary statistics for each group (where appropriate) and (b) an effect estimate and its precision (e.g. confidence/credible interval), ideally using structured tables or plots. | P 7 |
| Results of syntheses | 20a | For each synthesis, briefly summarise the characteristics and risk of bias among contributing studies. | N/A |
|  | 20b | Present results of all statistical syntheses conducted. If meta-analysis was done, present for each the summary estimate and its precision (e.g. confidence/credible interval) and measures of statistical heterogeneity. If comparing groups, describe the direction of the effect. | N/A |
|  | 20c | Present results of all investigations of possible causes of heterogeneity among study results. | N/A |
|  | 20d | Present results of all sensitivity analyses conducted to assess the robustness of the synthesized results. | N/A |
| Reporting biases | 21 | Present assessments of risk of bias due to missing results (arising from reporting biases) for each synthesis assessed. | N/A |
| Certainty of evidence | 22 | Present assessments of certainty (or confidence) in the body of evidence for each outcome assessed. | N/A |
| **DISCUSSION** | | |  |
| Discussion | 23a | Provide a general interpretation of the results in the context of other evidence. | P 8-9 |
|  | 23b | Discuss any limitations of the evidence included in the review. | P 9 |
|  | 23c | Discuss any limitations of the review processes used. | P 9 |
|  | 23d | Discuss implications of the results for practice, policy, and future research. | P 9-10 |
| **OTHER INFORMATION** | | |  |
| Registration and protocol | 24a | Provide registration information for the review, including register name and registration number, or state that the review was not registered. | P 4 |
|  | 24b | Indicate where the review protocol can be accessed, or state that a protocol was not prepared. | P 4 |
|  | 24c | Describe and explain any amendments to information provided at registration or in the protocol. | N/A |
| Support | 25 | Describe sources of financial or non-financial support for the review, and the role of the funders or sponsors in the review. | P 10 |
| Competing interests | 26 | Declare any competing interests of review authors. | P 10 |
| Availability of data, code and other materials | 27 | Report which of the following are publicly available and where they can be found: template data collection forms; data extracted from included studies; data used for all analyses; analytic code; any other materials used in the review. | P 9 |

*From:*  Page MJ, McKenzie JE, Bossuyt PM, Boutron I, Hoffmann TC, Mulrow CD, et al. The PRISMA 2020 statement: an updated guideline for reporting systematic reviews. BMJ 2021;372:n71. doi: 10.1136/bmj.n71

For more information, visit: <http://www.prisma-statement.org/>

**Data S2.**

Complete Search Strategy by Database and Grey Literature Search

**CINAHL via EBSCOhost (1936 - Present)**

Search conducted on 16 November, 2023

| **Search** | **Query** | **Records retrieved** |
| --- | --- | --- |
| S1 | (MH "Amenorrhea+") | 1,801 |
| S2 | amenorrh#e* | 2,998 |
| S3 | "functional hypothalamic amenorrh#e*" | 62 |
| S4 | ((menstrua* or menses) N3 (disrupt* or disturb* or dysfunction* or stop* or absent or absence or irregular* or status or disappear*)) | 1,843 |
| S5 | S1 OR S2 OR S3 OR S4 | 4,719 |
| S6 | (MH "Cardiovascular Diseases") OR (MH "Heart Diseases+") OR (MH "Arrhythmia+") OR (MH "Heart Failure+") OR (MH "Heart Valve Diseases+") OR (MH "Myocardial Diseases+") OR (MH "Myocardial Ischemia+") | 370,139 |
| S7 | (MH "Coronary Disease") | 25,648 |
| S8 | "pericardial disease*" | 241 |
| S9 | ((cardio* N4 disease*) or (heart N4 disease*) or (cardiac N4 disease*)) | 177,001 |
| S10 | (cardio* N4 (risk* or train* or health* or dysfunction* or impair* or function* or complication* or effect* or factor* or biomarker*)) | 123,757 |
| S11 | (cardiac N4 (train* or health* or dysfunction* or impair* or function* or complication* or factor* or biomarker*)) | 28,695 |
| S12 | (heart N4 (health* or function* or dysfunction* or complication*)) | 34,963 |
| S13 | ("vascular function*" or "vascular dysfunction*" or "endothelial dysfunction") | 8,147 |
| S14 | ("coronary arter* disease*" or atherosclerosis or "arter* hard*") | 49,758 |
| S15 | ("cardio* arrhythmia*" or "heart arrhythmia*" or "cardiac arrhythmia*" or "abnormal cardi* rhythm*" or "abnormal heart rhythm*" or "abnormal heart beat*" or "irregular cardi* rhythm*" or "irregular heart beat*" or "irregular heart rhythm*") | 3,807 |
| S16 | ("cardio* failure" or "heart failure" or "cardiac failure") | 80,596 |
| S17 | ("heart valve* disease*" or "cardiac valve* disease*" or "aortic stenosis" or "mitral valve insufficienc*" or "mitral valve prolapse") | 14,800 |
| S18 | ("aort* disease*" or "aortic aneurysm*") | 12,626 |
| S19 | (cardiomyopath* or "heart muscle disease*" or "enlarged heart*" or "enlarged cardiac muscle*" or myocarditis or pericarditis or "heart muscle* inflam*" or "cardi* inflam*") | 28,366 |
| S20 | (MH "Thrombosis+") OR (MH "Venous Thrombosis+") | 42,061 |
| S21 | "deep vein thrombosis" | 5,472 |
| S22 | ("cerebrovascular disease*" or stroke#) | 148,162 |
| S23 | "arter* disease*" | 36,736 |
| S24 | ("myocardial infarction*" or "heart attack*" or "cardi* infarction*" or "coronary infarction*" or STEMI or NSTEMI or "N-STEMI") | 75,905 |
| S25 | (MH "Hypertension+") | 92,944 |
| S26 | (hypertens* or "blood pressure*") | 207,726 |
| S27 | S6 OR S7 OR S8 OR S9 OR S10 OR S11 OR S12 OR S13 OR S14 OR S15 OR S16 OR S17 OR S18 OR S19 OR S20 OR S21 OR S22 OR S23 OR S24 OR S25 OR S26 | 812,006 |
| S28 | (pharmacologic* or non-pharmacologic* or treatment* or therap*) | 2,536,786 |
| S29 | ("life style*" or lifestyle* or smok* or tobacco or diet* or nutrition or "healthy eating" or (weight N2 (loss* or losing or manag* or gain* or healthy)) or exercis* or "physical* activ*" or stress or counsel*) | 1,087,732 |
| S30 | (medication* or medicine* or drug# or supplement* or "hormon* therap*" or "estrogen replacement therap*" or "oral contraceptive*" or "birth control pill*" or "folic acid") | 1,471,733 |
| S31 | (anticoagulant* or "blood thinner*" or warfarin or heparin or antiplatelet or aspirin or "Angiotensin-converting enzyme inhibitor*" or "ACE inhibitor*" or (angiotension N2 ("receptor blocker*" or inhibitor*)) or "beta blocker*" or "calcium channel blocker*" or digitalis or diuretics or vasodilator* or Nitroglycerin or statins or "nicotinic acid*" or "cholesterol absorption inhibitor*") | 115,853 |
| S32 | (MH "Drug Therapy") | 16,601 |
| S33 | (MH "Life Style+") OR (MH "Life Style, Sedentary+") | 279,924 |
| S34 | (MH "Life Style Changes") | 14,645 |
| S35 | "lipid profile*" | 9,127 |
| S36 | "flow-mediated dilation" | 1,284 |
| S37 | S28 OR S29 OR S30 OR S31 OR S32 OR S33 OR S34 OR S35 OR S36 | 3,775,709 |
| S38 | (MH "Exercise+") OR (MH "Running+") OR (MH "Walking+") OR (MH "Muscle Strengthening+") OR (MH "Upper Extremity Exercises+") OR (MH "Physical Activity") OR (MH "Physical Performance") OR (MH "Physical Fitness+") OR (MH "Sports+") OR (MH "Animal Sports+") OR (MH "Aquatic Sports+") OR (MH "Athletic Training+") OR (MH "Contact Sports+") OR (MH "Racquet Sports+") OR (MH "Skating+") OR (MH "Skiing+") OR (MH "Sports for Persons with Disabilities+") OR (MH "Team Sports+") OR (MH "Winter Sports+") | 261,586 |
| S39 | ("physical activity" or "physically active") | 98,073 |
| S40 | (exercis* or sport* or walk* or run or runs or runner* or running or jog* or swim* or dive or diving or bike* or biking or bicycl* or "physical fitness" or "physically fit" or "resistance training" or "endurance training" or "interval training" or HIIT or yoga or "tai chi" or "tai ji" or pilates or dance* or dancing or "weight lifting" or "weight lift" or badminton or tennis or squash or bowling or football or rugby or netball or basketball or soccer or hockey or skiing or boxing or golf* or ((horse* or equine) N2 (ride* or riding))) | 434,900 |
| S41 | (MH "Athletes+") | 35,274 |
| S42 | athlet* | 81,092 |
| S43 | S38 OR S39 OR S40 OR S41 OR S42 | 519,431 |
| S44 | S5 AND S27 AND S37 AND S43 | 76 |

**Cochrane Library via Wiley (1992 - Present)**

Search conducted on 16 November, 2023

| **Search** | **Query** | **Records retrieved** |
| --- | --- | --- |
| #1 | [mh ^amenorrhea] | 481 |
| #2 | amenorrh?e*:ti,ab,kw | 2981 |
| #3 | ((menstrua* or menses) NEAR/3 (disrupt* or disturb* or dysfunction* or stop* or absent or absence or irregular* or status or disappear*)):ti,ab,kw | 1498 |
| #4 | #1 or #2 or #3 | 4232 |
| #5 | [mh ^"cardiovascular diseases"] or [mh "heart valve diseases"] or [mh "arrhythmias, cardiac"] or [mh cardiomyopathies] or [mh "heart failure"] or [mh "heart valve diseases"] | 42799 |
| #6 | [mh ^"coronary artery disease"] | 9509 |
| #7 | (pericardial NEXT disease*):ti,ab,kw | 35 |
| #8 | ((cardio* or heart or cardiac) NEAR/4 disease*):ti,ab,kw | 69091 |
| #9 | (cardio* NEAR/4 (risk* or train* or health* or dysfunction* or impair* or function* or complication* or effect* or factor* or biomarker*)):ti,ab,kw | 57242 |
| #10 | (cardiac NEAR/4 (train* or health* or dysfunction* or impair* or function* or complication* or factor* or biomarker*)):ti,ab,kw | 13221 |
| #11 | (heart NEAR/4 (health* or function* or dysfunction* or complication*)):ti,ab,kw | 21230 |
| #12 | ((vascular NEXT (function* or dysfunction*)) or "endothelial dysfunction"):ti,ab,kw | 6175 |
| #13 | ((coronary NEXT arter* NEXT disease*) or atherosclerosis or (arter* NEXT hard*)):ti,ab,kw | 29782 |
| #14 | (((cardio* or heart or cardiac) NEXT arrhythmia*) or (abnormal NEXT cardi* NEXT rhythm*) or ((abnormal or irregular) NEXT (heart or cardi*) NEXT rhythm*) or ((abnormal or irregular) NEXT heart NEXT beat*)):ti,ab,kw | 4576 |
| #15 | ((cardio* or heart or cardiac) NEXT failure):ti,ab,kw | 36600 |
| #16 | ((cardiac or heart) NEXT valve* NEXT disease*):ti,ab,kw | 741 |
| #17 | ("aortic stenosis" or ("mitral valve" NEXT (insufficienc* or prolapse))):ti,ab,kw | 1888 |
| #18 | (aort* NEXT (disease* or aneurysm*)):ti,ab,kw | 2367 |
| #19 | [mh ^endocarditis] | 112 |
| #20 | (cardiomyopath* or ("heart muscle" NEXT disease*) or (enlarged NEXT (heart* or "cardiac muscle")) or myocarditis or pericarditis or (("heart muscle" or cardi*) NEXT inflam*)):ti,ab,kw | 6950 |
| #21 | [mh "arterial occlusive disease"] or [mh arteriosclerosis] | 17214 |
| #22 | [mh "cerebrovascular disorders"] or [mh "ischemic stroke"] | 22975 |
| #23 | [mh thrombosis] or [mh "venous thrombosis"] | 6902 |
| #24 | deep vein thrombosis:ti,ab,kw | 5819 |
| #25 | ((cerebrovascular NEXT disease*) or stroke?):ti,ab,kw | 72300 |
| #26 | (arter* NEXT disease*):ti,ab,kw | 26248 |
| #27 | [mh ^"myocardial infarction"] | 13141 |
| #28 | (((myocardial or cardi* or coronary) NEXT infarction*) or (heart NEXT attack*) or STEMI or NSTEMI or "N-STEMI"):ti,ab,kw | 36786 |
| #29 | [mh hypertension] | 26701 |
| #30 | (hypertensi* or "blood pressure"):ti,ab,kw | 151688 |
| #31 | {or #5-#30} | 352851 |
| #32 | (pharmacologic* or non-pharmacologic* or treatment* or therap*):ti,ab,kw | 1252508 |
| #33 | ((life NEXT style*) or lifestyle* or smok* or tobacco or diet* or nutrition or "healthy eating" or (weight NEAR/2 (loss* or losing or manag* or gain* or healthy)) or exercis* or (physical* NEXT activ*) or stress or counsel*):ti,ab,kw | 391970 |
| #34 | (medication* or medicine* or drug? or supplement* or ((hormon* or "estrogen replacement") NEXT therap*) or (oral NEXT contraceptive*) or ("birth control" NEXT pill*) or "folic acid"):ti,ab,kw | 869868 |
| #35 | (anticoagulant* or (blood NEXT thinner*) or warfarin or heparin or antiplatelet or aspirin or "Angiotensin-converting enzyme inhibitor" or "ACE inhibitor" or (angiotension NEAR/2 ((receptor NEXT blocker*) or inhibitor*)) or ((beta or "calcium channel") NEXT blocker*) or digitalis or diuretics or vasodilator* or Nitroglycerin or statins or "nicotinic acid" or "cholesterol absorption inhibitor"):ti,ab,kw | 74521 |
| #36 | [mh ^"drug therapy"] | 14826 |
| #37 | [mh ^"life style"] or [mh ^"healthy lifestyle"] or [mh ^"diet, healthy"] or [mh ^"sedentary behaviour"] | 5656 |
| #38 | lipid profile:ti,ab,kw | 9230 |
| #39 | flow-mediated dilation:ti,ab,kw | 2103 |
| #40 | {or #32-#39} | 1542268 |
| #41 | [mh exercise] or [mh ^"physical conditioning, human"] or [mh ^running] or [mh ^walking] | 38702 |
| #42 | [mh ^"tai ji"] or [mh ^yoga] | 1709 |
| #43 | [mh sports] or [mh "athletic performance"] or [mh "racquet sports"] or [mh "snow sports"] or [mh "water sports"] or [mh swimming] | 21039 |
| #44 | ("physical activity" or "physically active"):ti,ab,kw | 44064 |
| #45 | (exercis* or sport* or walk* or run or runs or runner* or running or jog* or swim* or dive or diving or bike* or biking or bicycl* or "physical fitness" or "physically fit" or "resistance training" or "endurance training" or "interval training" or HIIT or yoga or "tai chi" or "tai ji" or pilates or dance* or dancing or "weight lifting" or "weight lift" or badminton or tennis or squash or bowling or football or rugby or netball or basketball or soccer or hockey or skiing or boxing or golf* or ((horse* or equine) NEAR/2 (ride* or riding))):ti,ab,kw | 194672 |
| #46 | [mh athletes] | 1489 |
| #47 | athlet*:ti,ab,kw | 12683 |
| #48 | {or #41-#47} | 213449 |
| #49 | #4 and #31 and #40 and #48 | 86 |

**Embase <1974 to 2023 November 14>**

Search conducted on 14 November, 2023

| **Search** | **Query** | **Records retrieved** |
| --- | --- | --- |
| 1 | exp "amenorrhea and oligomenorrhea"/ | 35716 |
| 2 | amenorrh?e*.mp. | 32836 |
| 3 | functional hypothalamic amenorrh?e*.mp. | 304 |
| 4 | ((menstrua* or menses) adj3 (disrupt* or disturb* or dysfunction* or stop* or absent or absence or irregular* or status or disappear*)).mp. | 14937 |
| 5 | 1 or 2 or 3 or 4 | 47333 |
| 6 | cardiovascular disease/ or exp heart disease/ | 2449320 |
| 7 | exp heart arrhythmia/ | 607949 |
| 8 | exp cardiomyopathy/ | 178162 |
| 9 | exp heart failure/ | 646830 |
| 10 | exp valvular heart disease/ | 186851 |
| 11 | exp coronary artery disease/ | 348536 |
| 12 | pericardial disease*.mp. | 4092 |
| 13 | ((cardio* adj4 disease*) or (heart adj4 disease*) or (cardiac adj4 disease*)).mp. | 1021934 |
| 14 | (cardio* adj4 (risk* or train* or health* or dysfunction* or impair* or function* or complication* or effect* or factor* or biomarker*)).mp. | 598248 |
| 15 | (cardiac adj4 (train* or health* or dysfunction* or impair* or function* or complication* or factor* or biomarker*)).mp. | 180413 |
| 16 | (heart adj4 (health* or function* or dysfunction* or complication*)).mp. | 227771 |
| 17 | ("vascular function*" or "vascular dysfunction*" or "endothelial dysfunction").mp. | 84705 |
| 18 | ("coronary arter* disease*" or atherosclerosis or "arter* hard*").mp. | 537018 |
| 19 | ("cardio* arrhythmia*" or "heart arrhythmia*" or "cardiac arrhythmia*" or "abnormal cardi* rhythm*" or "abnormal heart rhythm*" or "abnormal heart beat*" or "irregular cardi* rhythm*" or "irregular heart beat*" or "irregular heart rhythm*").mp. | 154233 |
| 20 | ("cardio* failure" or "heart failure" or "cardiac failure").mp. | 518687 |
| 21 | ("heart valve* disease*" or "cardiac valve* disease*" or "aortic stenosis" or "mitral valve insufficienc*" or "mitral valve prolapse").mp. | 57150 |
| 22 | ("aort* disease*" or "aortic aneurysm*").mp. | 70515 |
| 23 | endocarditis/ | 25391 |
| 24 | (cardiomyopath* or "heart muscle disease*" or "enlarged heart*" or "enlarged cardiac muscle*" or myocarditis or pericarditis or "heart muscle* inflam*" or "cardi* inflam*").mp. | 252288 |
| 25 | exp atherosclerosis/ or exp peripheral arterial disease/ | 255989 |
| 26 | cerebrovascular disease/ | 69624 |
| 27 | exp ischemic stroke/ | 30927 |
| 28 | exp thrombosis/ or exp vein thrombosis/ | 437909 |
| 29 | deep vein thrombosis.mp. | 89538 |
| 30 | ("cerebrovascular disease*" or stroke?).mp. | 644509 |
| 31 | arter* disease*.mp. | 368289 |
| 32 | exp heart infarction/ | 450137 |
| 33 | ("myocardial infarction*" or "heart attack*" or "cardi* infarction*" or "coronary infarction*" or STEMI or NSTEMI or "N-STEMI").mp. | 357990 |
| 34 | exp hypertension/ | 939317 |
| 35 | (hypertens* or "blood pressure*").mp. | 1596480 |
| 36 | or/6-35 | 4820358 |
| 37 | (pharmacologic* or non-pharmacologic* or treatment* or therap*).mp. | 14364592 |
| 38 | ("life style*" or lifestyle* or smok* or tobacco or diet* or nutrition or "healthy eating" or (weight adj2 (loss* or losing or manag* or gain* or healthy)) or exercis* or "physical* activ*" or stress or counsel*).mp. | 4735075 |
| 39 | (medication* or medicine* or drug? or supplement* or "hormon* therap*" or "estrogen replacement therap*" or "oral contraceptive*" or "birth control pill?" or "folic acid").mp. | 14735426 |
| 40 | (anticoagulant* or "blood thinner*" or warfarin or heparin or antiplatelet or aspirin or "Angiotensin-converting enzyme inhibitor*" or "ACE inhibitor*" or (angiotension adj2 ("receptor blocker*" or inhibitor*)) or "beta blocker*" or "calcium channel blocker*" or digitalis or diuretics or vasodilator* or Nitroglycerin or statins or "nicotinic acid*" or "cholesterol absorption inhibitor*").mp. | 855739 |
| 41 | drug therapy/ | 954152 |
| 42 | exp lifestyle/ | 166778 |
| 43 | lifestyle and related phenomena/ or lifestyle modification/ | 53634 |
| 44 | healthy diet/ | 7448 |
| 45 | lipid profile.mp. | 48849 |
| 46 | flow-mediated dilation.mp. | 7417 |
| 47 | or/37-46 | 22758488 |
| 48 | exp exercise/ or exp continuous training/ or exp high intensity exercise/ or exp interval training/ or exp moderate intensity exercise/ or exp muscle exercise/ or exp resistance training/ | 437434 |
| 49 | fitness/ | 42792 |
| 50 | exp physical activity/ or exp climbing/ or exp running/ or exp walking/ or exp weight bearing/ | 542085 |
| 51 | exp sport/ or exp athletics/ or exp ball sport/ or exp climbing sport/ or exp combat sport/ or exp disabled sport/ or exp racquet sport/ or exp water sport/ or exp winter sport/ or exp yoga/ | 214734 |
| 52 | ("physical activity" or "physically active").mp. | 298792 |
| 53 | (exercis* or sport* or walk* or run or runs or runner* or running or jog* or swim* or dive or diving or bike* or biking or bicycl* or "physical fitness" or "physically fit" or "resistance training" or "endurance training" or "interval training" or HIIT or yoga or "tai chi" or "tai ji" or pilates or dance* or dancing or "weight lifting" or "weight lift" or badminton or tennis or squash or bowling or football or rugby or netball or basketball or soccer or hockey or skiing or boxing or golf* or ((horse* or equine) adj2 (ride* or riding))).mp. | 1370996 |
| 54 | exp athlete/ or exp ball sports athlete/ or exp combat sports athlete/ or exp disabled athlete/ | 78387 |
| 55 | athlet*.mp. | 119906 |
| 56 | or/48-55 | 1704592 |
| 57 | 5 and 36 and 47 and 56 | 722 |
| 58 | (conference or editorial or opinion or letter or note or book).pt. | 8777100 |
| 59 | 57 not 58 | 556 |

**Ovid MEDLINE ALL <1946 to November 14, 2023>**

Search conducted on 14 November, 2023

| **Search Number** | **Query** | **Records retrieved** |
| --- | --- | --- |
| 1 | amenorrhea/ | 10194 |
| 2 | amenorrh?e*.mp. | 19702 |
| 3 | functional hypothalamic amenorrh?e*.mp. | 216 |
| 4 | ((menstrua* or menses) adj3 (disrupt* or disturb* or dysfunction* or stop* or absent or absence or irregular* or status or disappear*)).mp. | 13305 |
| 5 | 1 or 2 or 3 or 4 | 30828 |
| 6 | cardiovascular diseases/ or exp heart diseases/ or exp arrhythmias, cardiac/ or exp cardiomyopathies/ or exp heart failure/ or exp heart valve diseases/ | 1436315 |
| 7 | Coronary Artery Disease/ | 77044 |
| 8 | pericardial disease*.mp. | 1334 |
| 9 | ((cardio* adj4 disease*) or (heart adj4 disease*) or (cardiac adj4 disease*)).mp. | 656546 |
| 10 | (cardio* adj4 (risk* or train* or health* or dysfunction* or impair* or function* or complication* or effect* or factor* or biomarker*)).mp. | 325952 |
| 11 | (cardiac adj4 (train* or health* or dysfunction* or impair* or function* or complication* or factor* or biomarker*)).mp. | 116914 |
| 12 | (heart adj4 (health* or function* or dysfunction* or complication*)).mp. | 67503 |
| 13 | ("vascular function*" or "vascular dysfunction*" or "endothelial dysfunction").mp. | 45607 |
| 14 | ("coronary arter* disease*" or atherosclerosis or "arter* hard*").mp. | 276130 |
| 15 | ("cardio* arrhythmia*" or "heart arrhythmia*" or "cardiac arrhythmia*" or "abnormal cardi* rhythm*" or "abnormal heart rhythm*" or "abnormal heart beat*" or "irregular cardi* rhythm*" or "irregular heart beat*" or "irregular heart rhythm*").mp. | 20529 |
| 16 | ("cardio* failure" or "heart failure" or "cardiac failure").mp. | 269137 |
| 17 | ("heart valve* disease*" or "cardiac valve* disease*" or "aortic stenosis" or "mitral valve insufficienc*" or "mitral valve prolapse").mp. | 77872 |
| 18 | ("aort* disease*" or "aortic aneurysm*").mp. | 84206 |
| 19 | Endocarditis/ | 11885 |
| 20 | (cardiomyopath* or "heart muscle disease*" or "enlarged heart*" or "enlarged cardiac muscle*" or myocarditis or pericarditis or "heart muscle* inflam*" or "cardi* inflam*").mp. | 155045 |
| 21 | exp arterial occlusive diseases/ or exp arteriosclerosis/ | 265427 |
| 22 | exp cerebrovascular disorders/ or exp ischemic stroke/ | 433091 |
| 23 | exp thrombosis/ or exp venous thrombosis/ | 152017 |
| 24 | deep vein thrombosis.mp. | 21656 |
| 25 | ("cerebrovascular disease*" or stroke?).mp. | 405939 |
| 26 | arter* disease*.mp. | 202192 |
| 27 | exp Myocardial Infarction/ | 195282 |
| 28 | ("myocardial infarction*" or "heart attack*" or "cardi* infarction*" or "coronary infarction*" or STEMI or NSTEMI or "N-STEMI").mp. | 286670 |
| 29 | exp Hypertension/ | 320119 |
| 30 | (hypertens* or "blood pressure*").mp. | 911835 |
| 31 | or/6-30 | 3257884 |
| 32 | (pharmacologic* or non-pharmacologic* or treatment* or therap*).mp. | 10346675 |
| 33 | ("life style*" or lifestyle* or smok* or tobacco or diet* or nutrition or "healthy eating" or (weight adj2 (loss* or losing or manag* or gain* or healthy)) or exercis* or "physical* activ*" or stress or counsel*).mp. | 3341113 |
| 34 | (medication* or medicine* or drug? or supplement* or "hormon* therap*" or "estrogen replacement therap*" or "oral contraceptive*" or "birth control pill?" or "folic acid").mp. | 8078106 |
| 35 | (anticoagulant* or "blood thinner*" or warfarin or heparin or antiplatelet or aspirin or "Angiotensin-converting enzyme inhibitor*" or "ACE inhibitor*" or (angiotension adj2 ("receptor blocker*" or inhibitor*)) or "beta blocker*" or "calcium channel blocker*" or digitalis or diuretics or vasodilator* or Nitroglycerin or statins or "nicotinic acid*" or "cholesterol absorption inhibitor*").mp. | 570170 |
| 36 | Drug Therapy/ | 31167 |
| 37 | life style/ or healthy lifestyle/ or diet, healthy/ or sedentary behavior/ | 86406 |
| 38 | lipid profile.mp. | 29528 |
| 39 | flow-mediated dilation.mp. | 4258 |
| 40 | or/32-39 | 15651879 |
| 41 | exp exercise/ or physical conditioning, human/ or running/ or walking/ | 250337 |
| 42 | tai ji/ or yoga/ | 5259 |
| 43 | exp sports/ or exp athletic performance/ or exp racquet sports/ or exp snow sports/ or exp water sports/ or exp swimming/ | 218817 |
| 44 | ("physical activity" or "physically active").mp. | 157527 |
| 45 | (exercis* or sport* or walk* or run or runs or runner* or running or jog* or swim* or dive or diving or bike* or biking or bicycl* or "physical fitness" or "physically fit" or "resistance training" or "endurance training" or "interval training" or HIIT or yoga or "tai chi" or "tai ji" or pilates or dance* or dancing or "weight lifting" or "weight lift" or badminton or tennis or squash or bowling or football or rugby or netball or basketball or soccer or hockey or skiing or boxing or golf* or ((horse* or equine) adj2 (ride* or riding))).mp. | 1009462 |
| 46 | exp Athletes/ | 20973 |
| 47 | athlet*.mp. | 111793 |
| 48 | or/41-47 | 1100097 |
| 49 | 5 and 31 and 40 and 48 | 172 |

**Scopus (1976 - Present)**

Search conducted on 16 November, 2023

| **Query** | **Results Retrieved** |
| --- | --- |
| ( TITLE-ABS-KEY ( amenorrh?e* OR "functional hypothalamic amenorrh?e*" OR ( ( ( menstrua* OR menses ) W/3 ( disrupt* OR disturb* OR dysfunction* OR stop* OR absent OR absence OR irregular* OR status OR disappear* ) ) ) ) ) AND ( ( TITLE-ABS-KEY ( ( ( ( cardio* W/4 disease* ) OR ( heart W/4 disease* ) OR ( cardiac W/4 disease* ) ) ) OR ( ( cardio* W/4 ( risk* OR train* OR health* OR dysfunction* OR impair* OR function* OR complication* OR effect* OR factor* OR biomarker* ) ) ) OR ( ( cardiac W/4 ( train* OR health* OR dysfunction* OR impair* OR function* OR complication* OR factor* OR biomarker* ) ) ) OR ( ( heart W/4 ( health* OR function* OR dysfunction* OR complication* ) ) ) ) ) OR ( TITLE-ABS-KEY ( "pericardial disease*" OR ( ( "vascular function*" OR "vascular dysfunction*" OR "endothelial dysfunction" ) ) OR ( ( "coronary arter* disease*" OR atherosclerosis OR "arter* hard*" ) ) OR ( ( "cardio* arrhythmia*" OR "heart arrhythmia*" OR "cardiac arrhythmia*" OR "abnormal cardi* rhythm*" OR "abnormal heart rhythm*" OR "abnormal heart beat*" OR "irregular cardi* rhythm*" OR "irregular heart beat*" OR "irregular heart rhythm*" ) ) OR ( ( "cardio* failure" OR "heart failure" OR "cardiac failure" ) ) OR ( ( "heart valve* disease*" OR "cardiac valve* disease*" OR "aortic stenosis" OR "mitral valve insufficienc*" OR "mitral valve prolapse" ) ) OR ( ( "aort* disease*" OR "aortic aneurysm*" ) ) OR ( ( cardiomyopath* OR "heart muscle disease*" OR "enlarged heart*" OR "enlarged cardiac muscle*" OR myocarditis OR pericarditis OR "heart muscle* inflam*" OR "cardi* inflam*" ) ) OR ( "deep vein thrombosis" OR "venous thrombosis" OR thromboembolism* ) OR ( ( "cerebrovascular disease*" OR stroke? ) ) ) ) OR ( TITLE-ABS-KEY ( "arter* disease*" OR ( ( "myocardial infarction*" OR "heart attack*" OR "cardi* infarction*" OR "coronary infarction*" OR stemi OR nstemi OR "N-STEMI" ) ) OR ( ( hypertens* OR "blood pressure*" ) ) ) ) ) AND ( TITLE-ABS-KEY ( ( ( pharmacologic* OR non-pharmacologic* OR treatment* OR therap* ) ) OR ( ( "life style*" OR lifestyle* OR smok* OR tobacco OR diet* OR nutrition OR "healthy eating" OR ( weight W/2 ( loss* OR losing OR manag* OR gain* OR healthy ) ) OR exercis* OR "physical* activ*" OR stress OR counsel* ) ) OR ( ( medication* OR medicine* OR drug# OR supplement* OR "hormon* therap*" OR "estrogen replacement therap*" OR "oral contraceptive*" OR "birth control pill*" OR "folic acid" ) ) OR ( ( anticoagulant* OR "blood thinner*" OR warfarin OR heparin OR antiplatelet OR aspirin OR "Angiotensin-converting enzyme inhibitor*" OR "ACE inhibitor*" OR ( angiotension W/2 ( "receptor blocker*" OR inhibitor* ) ) OR "beta blocker*" OR "calcium channel blocker*" OR digitalis OR diuretics OR vasodilator* OR nitroglycerin OR statins OR "nicotinic acid*" OR "cholesterol absorption inhibitor*" ) ) OR "lipid profile*" OR "flow-mediated dilation" ) ) AND ( TITLE-ABS-KEY ( ( ( "physical activity" OR "physically active" ) ) OR ( ( exercis* OR sport* OR walk* OR run OR runs OR runner* OR running OR jog* OR swim* OR dive OR diving OR bike* OR biking OR bicycl* OR "physical fitness" OR "physically fit" OR "resistance training" OR "endurance training" OR "interval training" OR hiit OR yoga OR "tai chi" OR "tai ji" OR pilates OR dance* OR dancing OR "weight lifting" OR "weight lift" OR badminton OR tennis OR squash OR bowling OR football OR rugby OR netball OR basketball OR soccer OR hockey OR skiing OR boxing OR golf* OR ( ( horse* OR equine ) W/2 ( ride* OR riding ) ) ) ) OR athlet* ) ) AND ( LIMIT-TO ( DOCTYPE , "re" ) OR LIMIT-TO ( DOCTYPE , "ar" ) ) | 286 |

**SPORTDiscus via EBSCOhost**

Search modes - Find all my search terms

Search conducted on 16 November, 2023

| **Search Number** | **Query** | **Records retrieved** |
| --- | --- | --- |
| S1 | amenorrh#e* OR "functional hypothalamic amenorrh#e*" OR ( ((menstrua* or menses) N3 (disrupt* or disturb* or dysfunction* or stop* or absent or absence or irregular* or status or disappear*)) ) | 1,611 |
| S2 | ( ((cardio* N4 disease*) or (heart N4 disease*) or (cardiac N4 disease*)) ) OR ( (cardio* N4 (risk* or train* or health* or dysfunction* or impair* or function* or complication* or effect* or factor* or biomarker*)) ) OR ( (cardiac N4 (train* or health* or dysfunction* or impair* or function* or complication* or factor* or biomarker*)) ) OR ( (heart N4 (health* or function* or dysfunction* or complication*)) ) | 45,097 |
| S3 | "pericardial disease*" OR ( ("vascular function*" or "vascular dysfunction*" or "endothelial dysfunction") ) OR ( ("coronary arter* disease*" or atherosclerosis or "arter* hard*") ) OR ( ("cardio* arrhythmia*" or "heart arrhythmia*" or "cardiac arrhythmia*" or "abnormal cardi* rhythm*" or "abnormal heart rhythm*" or "abnormal heart beat*" or "irregular cardi* rhythm*" or "irregular heart beat*" or "irregular heart rhythm*") ) OR ( ("cardio* failure" or "heart failure" or "cardiac failure") ) OR ( ("heart valve* disease*" or "cardiac valve* disease*" or "aortic stenosis" or "mitral valve insufficienc*" or "mitral valve prolapse") ) OR ( ("aort* disease*" or "aortic aneurysm*") ) OR ( (cardiomyopath* or "heart muscle disease*" or "enlarged heart*" or "enlarged cardiac muscle*" or myocarditis or pericarditis or "heart muscle* inflam*" or "cardi* inflam*") ) OR ( "deep vein thrombosis" or "venous thrombosis" or thromboembolism* ) OR ( ("cerebrovascular disease*" or stroke#) ) | 41,814 |
| S4 | "arter* disease*" OR ( ("myocardial infarction*" or "heart attack*" or "cardi* infarction*" or "coronary infarction*" or STEMI or NSTEMI or "N-STEMI") ) OR ( (hypertens* or "blood pressure*") ) | 39,305 |
| S5 | S2 OR S3 OR S4 | 98,460 |
| S6 | ( (pharmacologic* or non-pharmacologic* or treatment* or therap*) ) OR ( ("life style*" or lifestyle* or smok* or tobacco or diet* or nutrition or "healthy eating" or (weight N2 (loss* or losing or manag* or gain* or healthy)) or exercis* or "physical* activ*" or stress or counsel*) ) OR ( (medication* or medicine* or drug# or supplement* or "hormon* therap*" or "estrogen replacement therap*" or "oral contraceptive*" or "birth control pill*" or "folic acid") ) OR ( (anticoagulant* or "blood thinner*" or warfarin or heparin or antiplatelet or aspirin or "Angiotensin-converting enzyme inhibitor*" or "ACE inhibitor*" or (angiotension N2 ("receptor blocker*" or inhibitor*)) or "beta blocker*" or "calcium channel blocker*" or digitalis or diuretics or vasodilator* or Nitroglycerin or statins or "nicotinic acid*" or "cholesterol absorption inhibitor*") ) OR "lipid profile*" OR "flow-mediated dilation" | 865,104 |
| S7 | ( ("physical activity" or "physically active") ) OR ( (exercis* or sport* or walk* or run or runs or runner* or running or jog* or swim* or dive or diving or bike* or biking or bicycl* or "physical fitness" or "physically fit" or "resistance training" or "endurance training" or "interval training" or HIIT or yoga or "tai chi" or "tai ji" or pilates or dance* or dancing or "weight lifting" or "weight lift" or badminton or tennis or squash or bowling or football or rugby or netball or basketball or soccer or hockey or skiing or boxing or golf* or ((horse* or equine) N2 (ride* or riding))) ) OR athlet* | 1,840,489 |
| S8 | S1 AND S5 AND S6 AND S7 | 56 |

**Grey literature search:**

**World Health Organization International Clinical Trials Registry Platform**

Search conducted on 1 May, 2024

Keywords: “amenorrhe* AND “cardiovascular or cardiovascular disease” AND “female athlet*” AND “intervention”

Results = 0

**Open Science Framework**

Search conducted on 1 May, 2024

Keywords: “amenorrhea” AND “cardiovascular or cardiovascular disease” AND “female athlete” AND “intervention”

Results = 0

**Web of Science Conference Proceedings Index**

Search conducted on 1 May, 2024

Keywords: amenorrhea, cardiovascular or cardiovascular disease, female athlete, exercise

Results=0

**ProQuest Dissertations and Theses Global**

Search conducted on 1 May, 2024

Keywords: “amenorrhe*” AND “female athlet* OR exercis*” AND “cardiovascular disease OR cardiovascular” AND “intervention”. Searched: “Anywhere except full text - NOFT”. Manuscript type: “select all”. Language “select all”.

Results = 0

| **Table S1**  *Studies Ineligible Following Full-Text Review* | |
| --- | --- |
| # | Citation and reason for exclusion |
| 1 | Cumming, DC, Cumming, CE. Estrogen replacement therapy and female athletes: current issues. Sports Med 2001; 31:1025-1031.*Reason for exclusion: Ineligible study design. This was a review, not a primary study.* |
| 2 | Grosman-Rimon L, Wright E, Freedman D,et al. Can improvement in hormonal and energy balance reverse cardiovascular risk factors in athletes with amenorrhea? Am J Physiol Heart Circ Physiol 2019;317(3):H487-H95.  *Reason for exclusion: Ineligible study design. This was a review, not a primary study.* |
| 3 | Hoch, AZ, Jurva, JW, Staton, MA, et al. Athletic amenorrhea and endothelial dysfunction. Wis Med J 2007;106(2).  *Reason for exclusion: Ineligible study design. Cohort study.* |
| 4 | Hoch, AZ, Pajewski, NM, Hoffmann, RG, et al. Possible relationship of folic acid supplementation and improved flow-mediated dilation in premenopausal, eumenorrheic athletic women. J Sports Sci Med 2009; 8(1): 123.  *Reason for exclusion: Ineligible participant characteristics. No women with amenorrhea.* |
| 5 | Hoch, AZ, Papanek, P, Szabo, A, et al. Folic acid supplementation improves vascular function in professional dancers with endothelial dysfunction. PM R 2011;3(11): 1005-1012.  *Reason for exclusion: Ineligible participant characteristics. Groups based on flow-mediated dilation %, not menstrual status.* |
| 6 | Zach KN, Smith Machin AL, Hoch AZ. Advances in management of the female athlete triad and eating disorders. Clin Sports Med. 2011;30(3):551-73.  *Reason for exclusion: Ineligible study design. This was a review, not a primary study.* |

#### **Table S2**

#### Critical Appraisal of Eligible Quasi-Experimental Study

| **Citation** | **Q1** | **Q2** | **Q3** | **Q4** | **Q5** | **Q6** | **Q7** | **Q8** | **Q9** | **Quality** |
| --- | --- | --- | --- | --- | --- | --- | --- | --- | --- | --- |
| Rickenlund et al. 2005. | Y | Y | Y | Y | N | Y | Y | Y | Y | Good |
| Hoch et al. 2010. | Y | Y | Y | Y | N | Y | Y | Y | Y | Good |

#### Y, yes; N, no

#### JBI critical appraisal checklist for quasi-experimental studies. 1. Is it clear in the study what is the ‘cause’ and what is the ‘effect’ (i.e. there is no confusion about which variable comes first)? 2. Were the participants included in any comparisons similar? 3. Were the participants included in any comparisons receiving similar treatment/care, other than the exposure or intervention of interest? 4. Was there a control group? 5. Were there multiple measurements of the outcome both pre and post the intervention/exposure? 6. Was follow up complete and if not, were differences between groups in terms of their follow up adequately described and analyzed? 7. Were the outcomes of participants included in any comparisons measured in the same way? 8. Were outcomes measured in a reliable way? 9. Was appropriate statistical analysis used?

#### **Table S3**

#### Critical Appraisal of Eligible Randomized Controlled Trial

| **Citation** | **Q1** | **Q2** | **Q3** | **Q4** | **Q5** | **Q6** | **Q7** | **Q8** | **Q9** | **Q10** | **Q11** | **Q12** | **Q13** | **Quality** |
| --- | --- | --- | --- | --- | --- | --- | --- | --- | --- | --- | --- | --- | --- | --- |
| Dadgostar et al. 2018. | Y | U | U | U | U | U | U | Y | Y | Y | U | N | U | Fair |

#### Y, yes; N, no; U, unsure

*JBI critical appraisal checklist for randomized controlled trial. 1. Was true randomization used for assignment of participants to treatment groups? 2. Was allocation to treatment groups concealed? 3. Were treatment groups similar at the baseline? 4. Were participants blind to treatment assignment? 5. Were those delivering treatment blind to treatment assignment? 6. Were outcomes assessors blind to treatment assignment? 7. Were treatment groups treated identically other than the intervention of interest? 8. Was follow up complete and if not, were differences between groups in terms of their follow up adequately described and analyzed? 9. Were participants analyzed in the groups to which they were randomized? 10. Were outcomes measured in the same way for treatment groups? 11. Were outcomes measured in a reliable way? 12. Was appropriate statistical analysis used? 13. Was the trial design appropriate, and any deviations from the standard RCT design (individual randomization, parallel groups) accounted for in the conduct and analysis of the trial*
